# Supplementary material for: Core-shell nanoscale coordination polymers combine chemotherapy and photodynamic therapy to potentiate checkpoint blockade cancer immunotherapy
Source: Nat Commun. 2016 Aug 17;7:12499. doi: 10.1038/ncomms12499 (PMC4992065; doi:10.1038/ncomms12499)
Supplement: Supplementary Information — Supplementary Figures 1-24, Supplementary Table 1, Supplementary Methods and Supplementary References. [file ncomms12499-s1.pdf]

Supplementary Figures

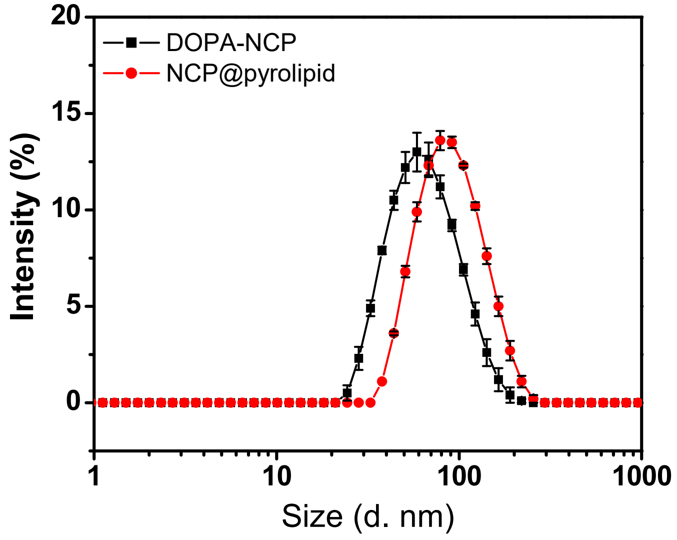

Supplementary Figure 1 Intensity-average of DOPA-NCP (in THF) and NCP@pyrolipid (redispersed in PBS) by DLS measurements (n=3).

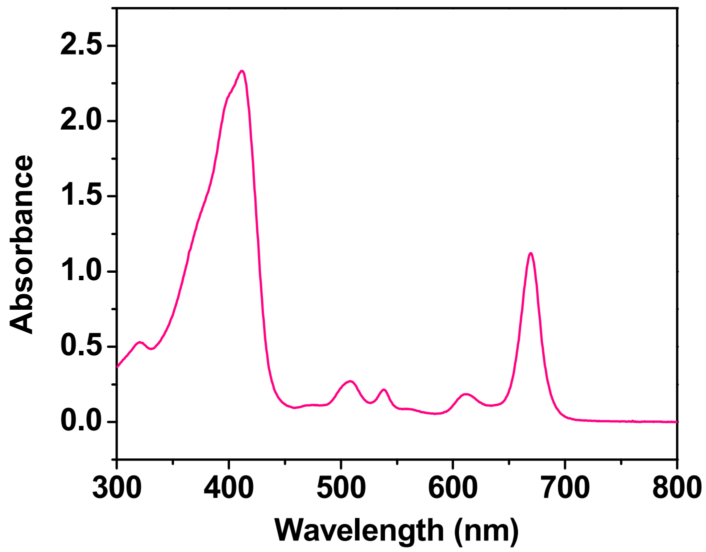

Supplementary Figure 2 UV-vis spectrum of NCP@pyrolipid dispersed in THF showing a broad Soret band around 400 nm and a distinct Q-band at 669 nm. When dispersed in

THF, the lipid bilayer of NCP@pyrolipid dissolved, and pyrolipid showed a broad Soret band around 400 nm and a distinct Q-band at 669 nm.

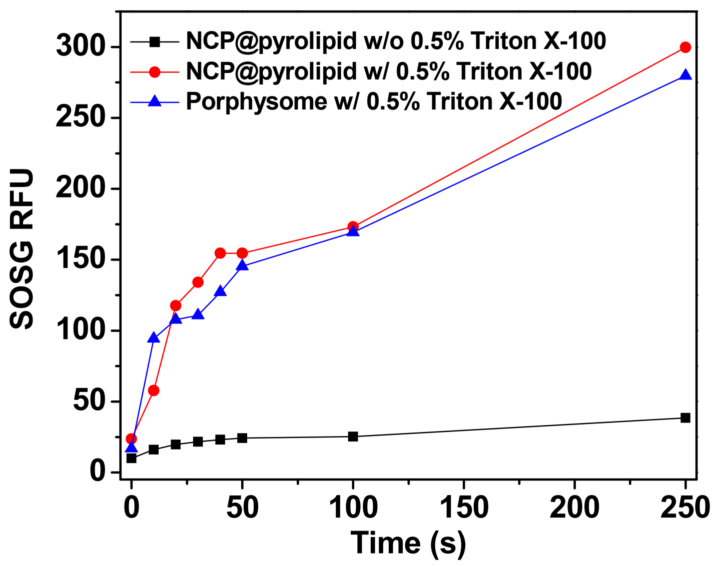

Supplementary Figure 3 Singlet oxygen generation of NCP@pyrolipid with and without Triton X-100 and porphysome with Triton X-100 upon light irradiation (670 nm, 100 mW/cm^2) determined by the singlet oxygen sensor green (SOSG) reagent. Porphysome was prepared by following the procedure reported by Zheng and coworkers. [1-4] When the lipid bilayer remains intact, the pyrolipid excited states are highly quenched, thereby generating very low amount of $^1O_2$. After adding Triton X-100 to NCP@pyrolipid and porphysome to disrupt the lipid bilayer, pyrolipid regained its fluorescence and the released species from both systems efficiently generated similar amounts of $^1O_2$.

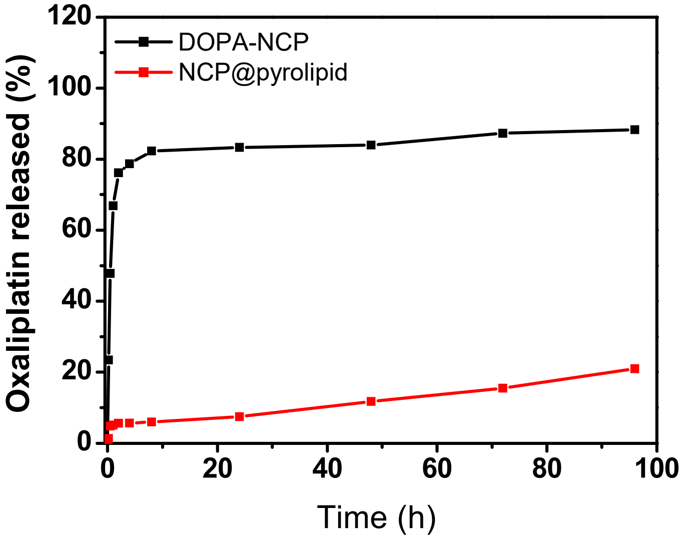

Supplementary Figure 4 Release profiles of oxaliplatin from DOPA-NCP and NCP@pyrolipid in PBS.

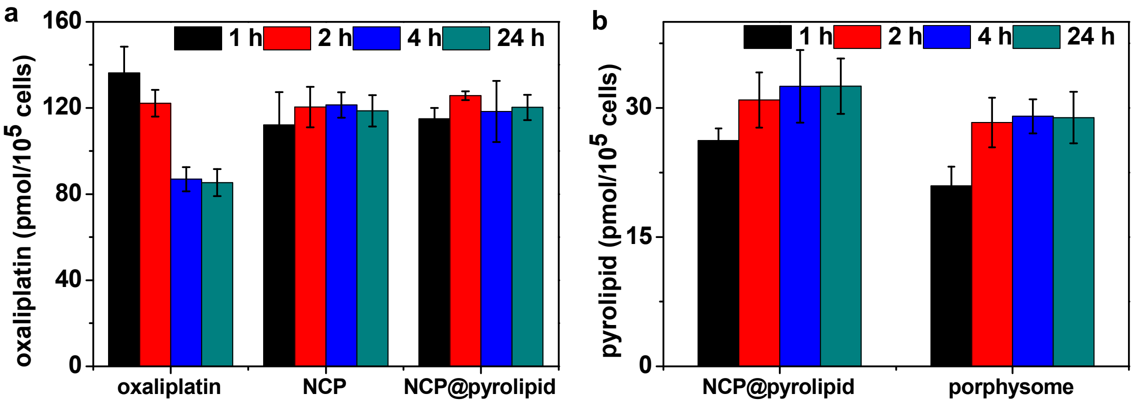

Supplementary Figure 5 Cellular uptake of NCP@pyrolipid in CT26 cells (n=3). Oxaliplatin (a) and pyrolipid (b) uptake amounts were determined by ICP-MS and spectrofluorophotometer, respectively. The cellular uptake of NCP@pyrolipid was rapid and nearly complete within 1 hour in terms of both oxaliplatin and pyrolipid, as evidenced by the stable concentrations of both components up to 24 hours. Cellular uptake of oxaliplatin and pyrolipid remained stable throughout the 24-hour experiment in NCP and porphysome formulations but decreased significantly for free oxaliplatin, suggesting that the internalization of NCP particles might lead to reduced efflux of oxaliplatin, potentially by changing the dynamics, porosity, and permeability of the cell membrane.

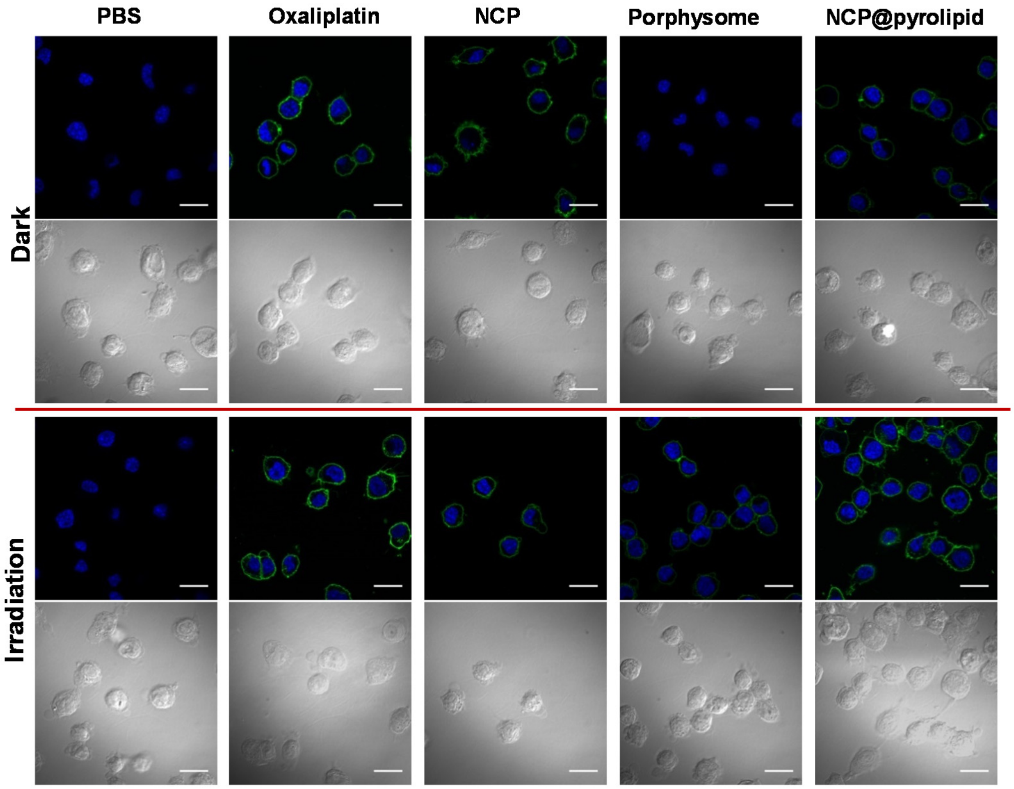

Supplementary Figure 6 Immunofluorescence microscopy of CRT expression on the cell surface of CT26 cells treated with PBS, free oxaliplatin, NCP, porphysome, and NCP@pyrolipid in the absence or presence of light irradiation (90 J/cm^2). Blue: DAPI stained nuclei; Green: Alexa Fluor 488-CRT antibody. Bar=20 μm.

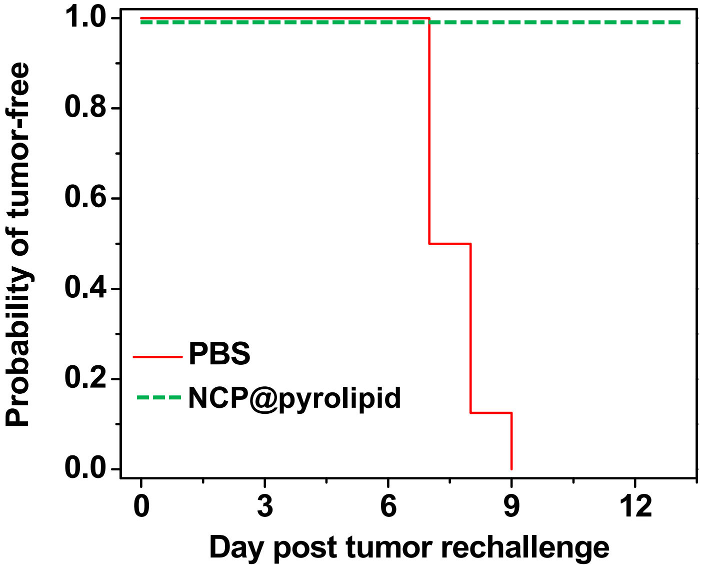

Supplementary Figure 7 Probability of tumor-free mice after tumor rechallenge (n=3). NCP@pyrolipid with irradiation-treated CT26 cells were efficiently vaccinated against live tumor cells. CT26 cells treated *in vitro* with NCP@pyrolipid and light irradiation were inoculated subcutaneously in BALB/c mice. After 7 days, mice were rechallenged with live CT26 cells.

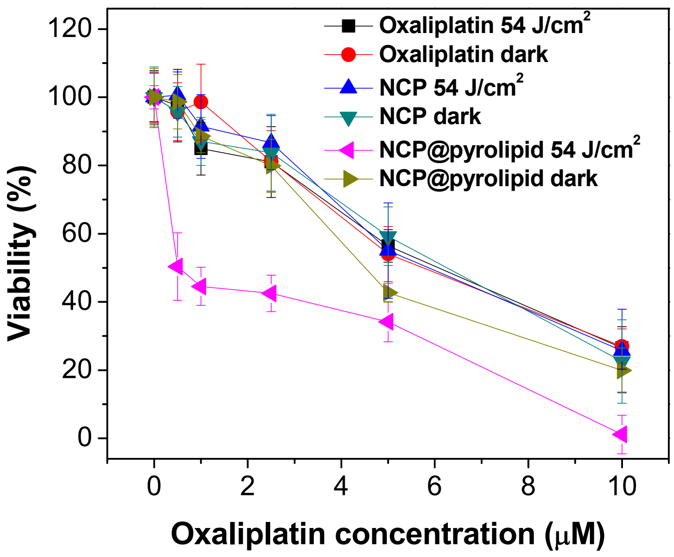

Supplementary Figure 8 Cell viability of CT26 cells treated with NCP@pyrolipid at different oxaliplatin doses (n=6).

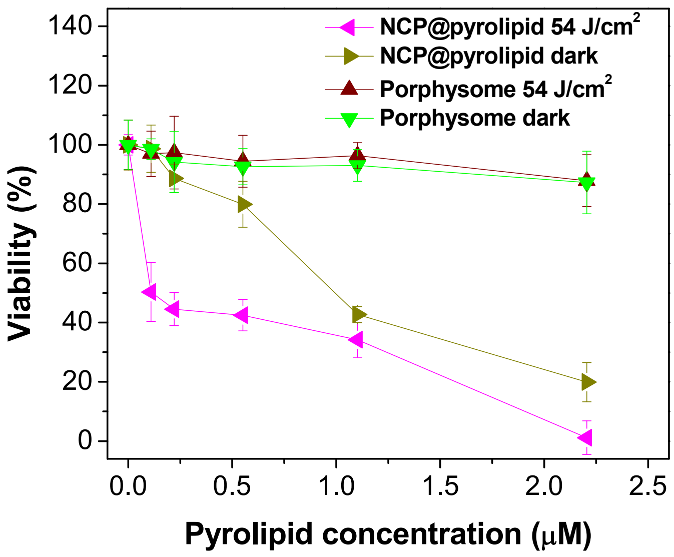

Supplementary Figure 9 Cell viability of CT26 cells treated with NCP@pyrolipid at different pyrolipid doses (n=6).

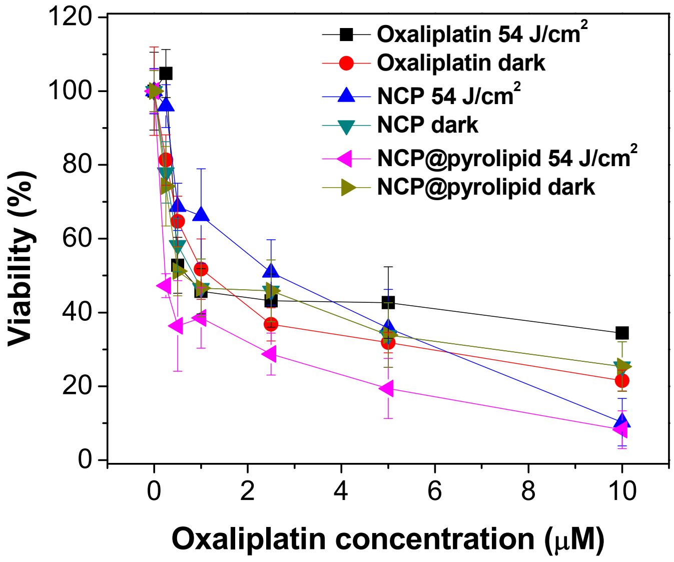

Supplementary Figure 10 Cell viability of HT29 cells treated with NCP@pyrolipid at different oxaliplatin doses (n=6).

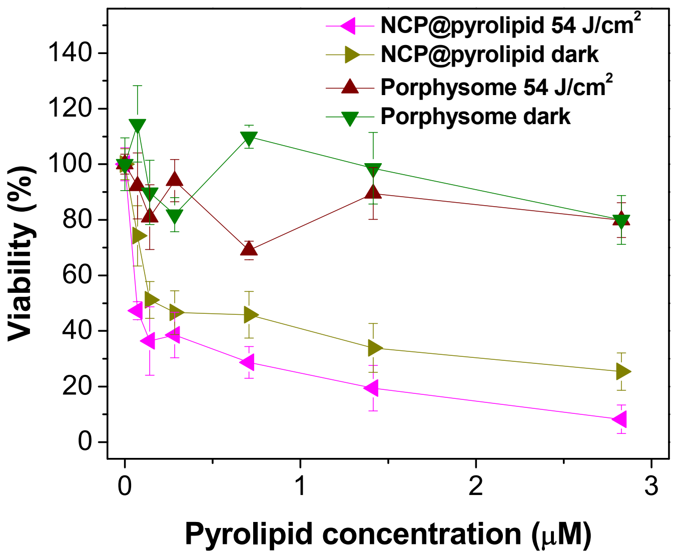

Supplementary Figure 11 Cell viability of HT29 cells treated with NCP@pyrolipid at different pyrolipid doses (n=6).

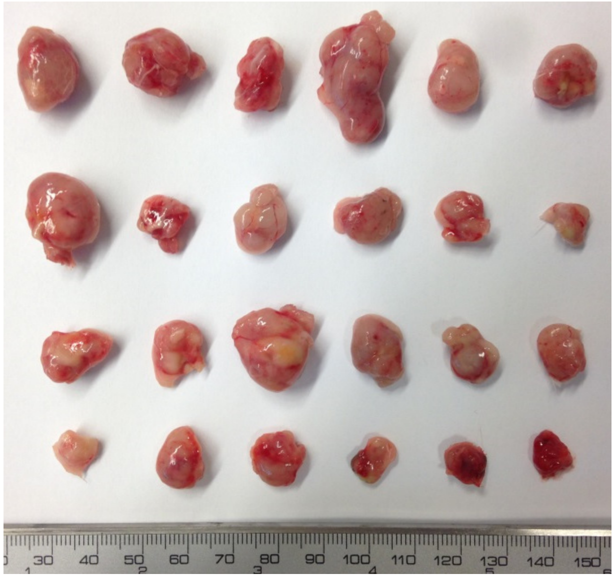

Supplementary Figure 12 *In vivo* anticancer activity of NCP@pyrolipid against CT26 tumor-bearing mice. Photographs of excised tumors at endpoint. From top to bottom: PBS w/ irradiation, NCP w/ irradiation, NCP@pyrolipid w/o irradiation, NCP@pyrolpid w/ irradiation.

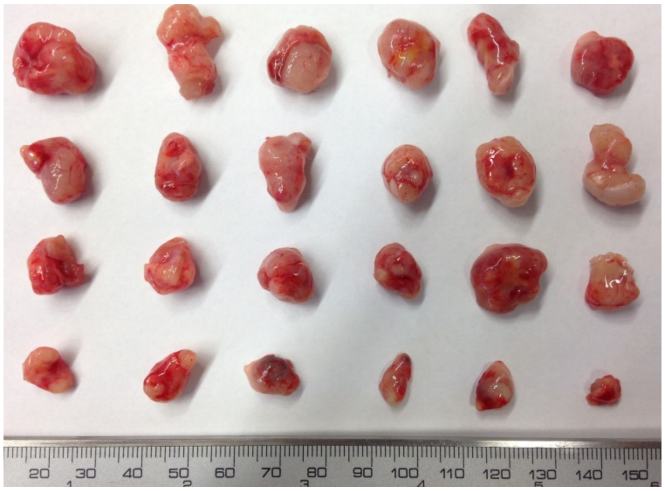

Supplementary Figure 13 *In vivo* anticancer activity of NCP@pyrolipid against HT29 tumor-bearing mice. Photographs of excised tumors at endpoint. From top to bottom: PBS

w/ irradiation, NCP w/ irradiation, NCP@pyrolipid w/o irradiation, NCP@pyrolipid w/ irradiation.

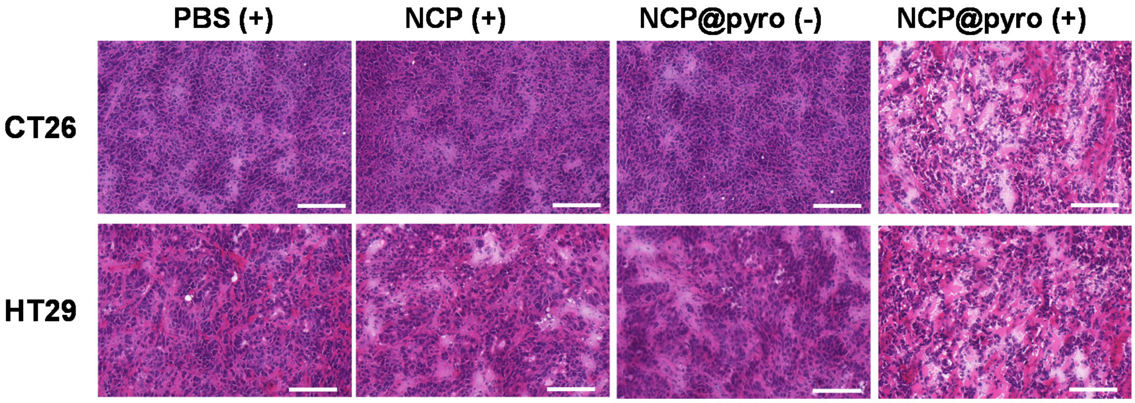

Supplementary Figure 14 H&E staining of tumor sections harvested from CT26 or HT29 tumor-bearing mice receiving PBS w/ irradiation, NCP w/ irradiation, NCP@pyrolipid w/o irradiation, and NCP@pyrolipid w/ irradiation. Bar=50 μm.

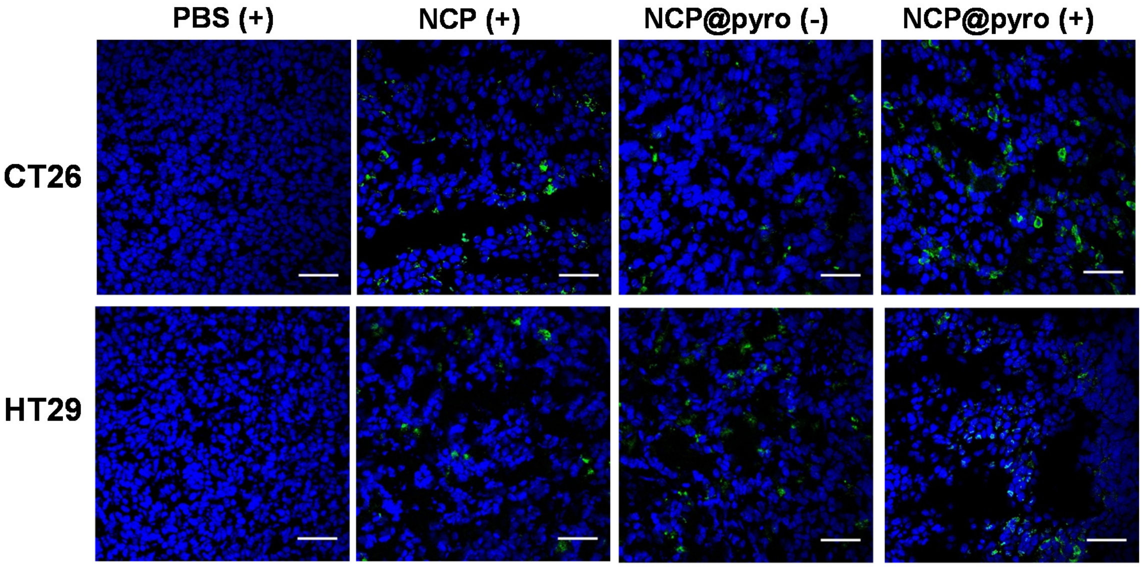

Supplementary Figure 15 Representative CLSM images of TUNEL assays of tumor tissues. DNA fragments in apoptotic cells were stained with fluorescein-conjugated deoxynucleotides (green), and the nuclei were stained with DAPI (blue). Bar=50 μm.

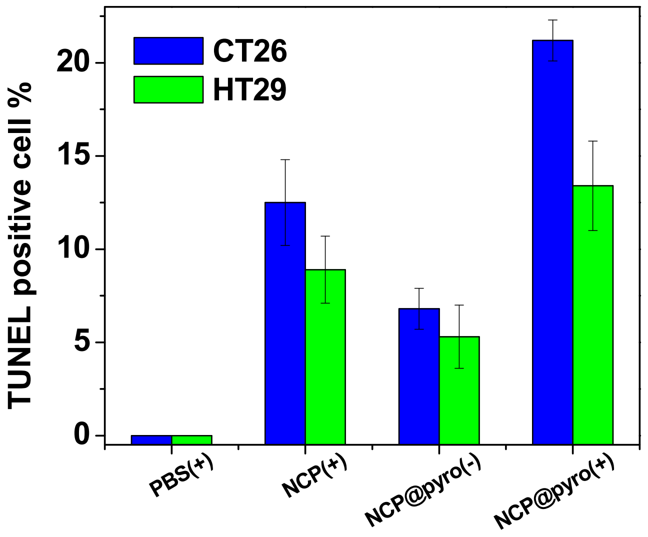

Supplementary Figure 16 Percentages of TUNEL-positive cells in tumor tissues (n=3).

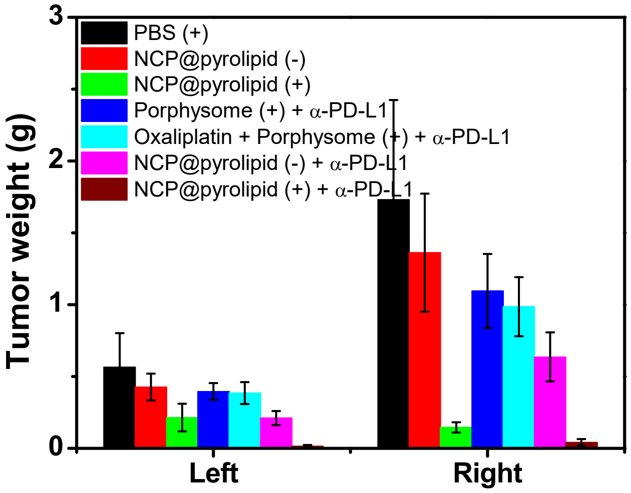

Supplementary Figure 17 Tumor weights of MC38 tumor-bearing mice receiving the treatment described in **Figures 6a and 6b** (n=6).

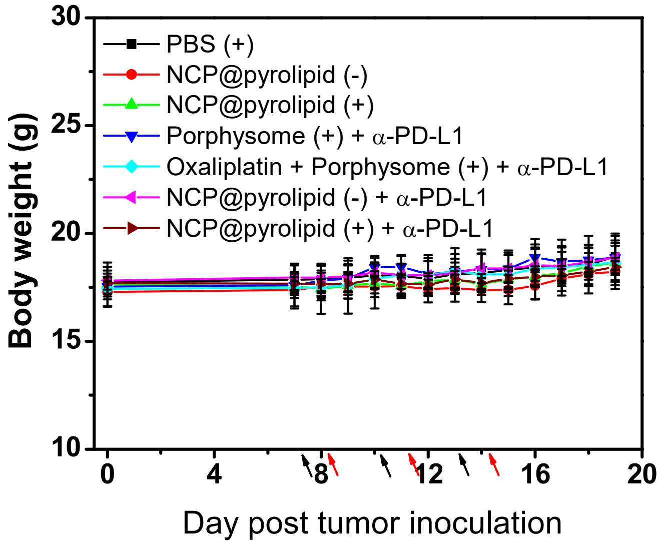

Supplementary Figure 18 Body weights of MC38 tumor-bearing mice receiving the treatment described in **Figures. 6a and 6b** (n=6).

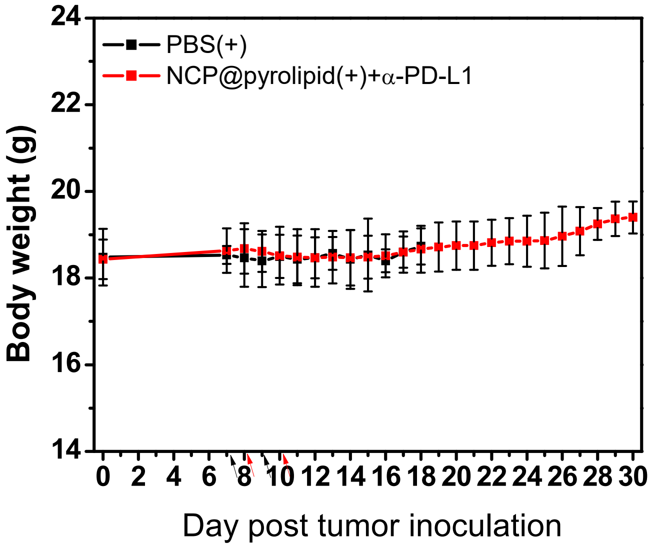

Supplementary Figure 19 Body weights of CT26 tumor-bearing mice receiving the treatment described in **Figures 6c and 6d** (n=6).

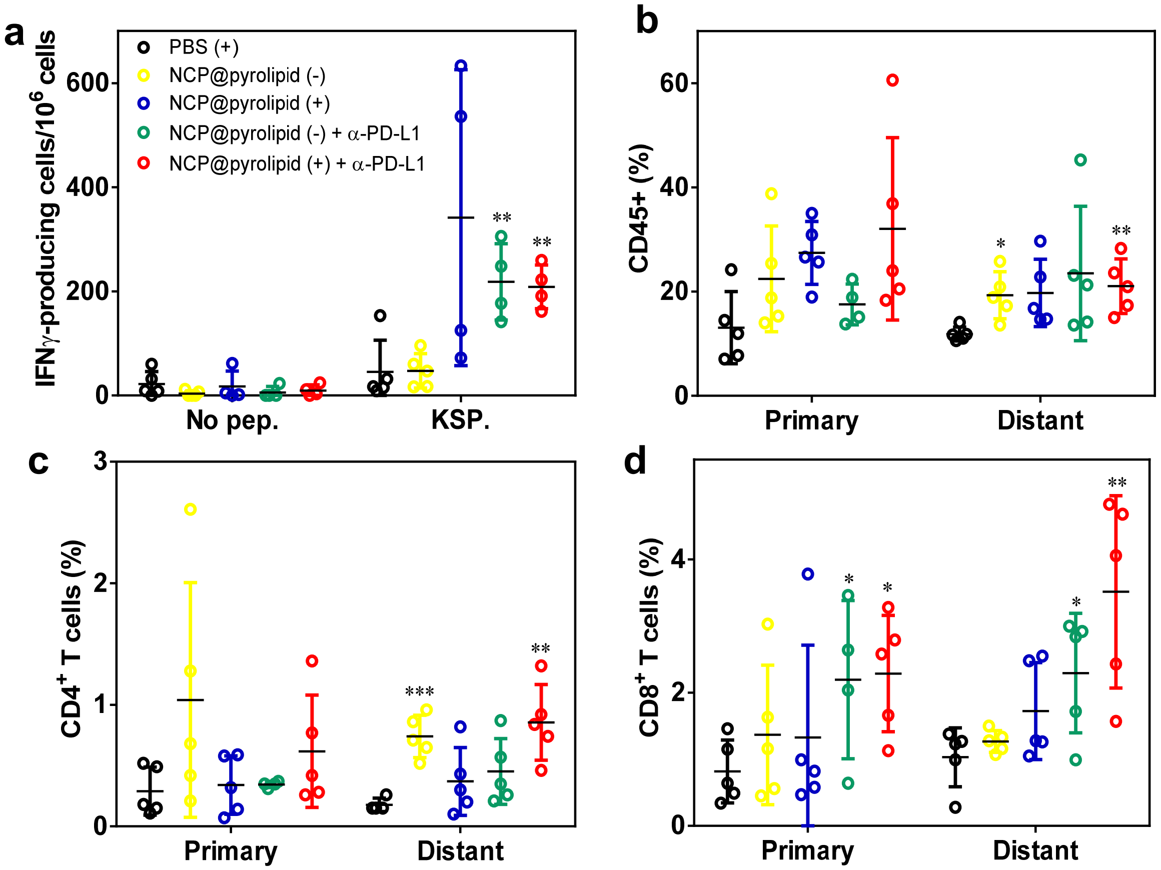

Supplementary Figure 20 (a) The number of antigen-specific IFN-γ producing T cells determined by ELISPOT assay. Percentages of tumor-infiltrating $CD45^+$ cells **(b)**, $CD4^+$ T cells **(c)** and $CD8^+$ T cells **(d)** of MC38 tumor-bearing mice treated as described in **Figures 6a and 6b**. The primary (right) and distant (left) tumors were collected for flow cytometry analysis. The cells were stained with $CD45^+PI^-$ **(b)**, $CD45^+CD3e^+CD4^+PI^-$ **(c)**, and $CD45^+CD3e^+CD8^+PI^-$ **(d)**, and gated from total tumor cells. *: $P < 0.05$ from control; **: $P < 0.01$ from control; ***: $P < 0.001$ from control.

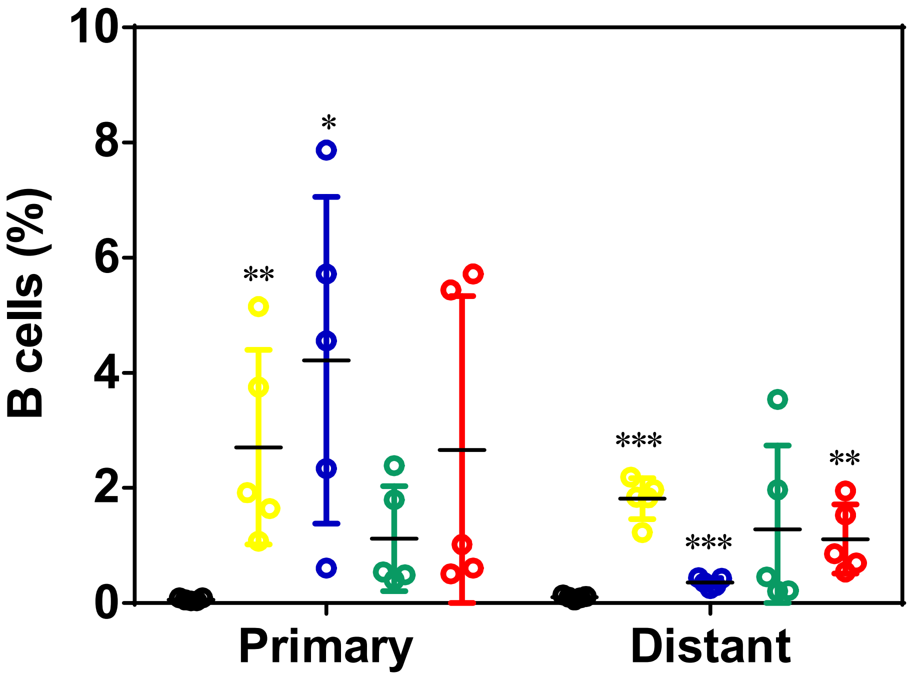

Supplementary Figure 21 Percentages of tumor-infiltrating B cells of MC38 tumor-bearing mice treated as described in **Figures 6a and 6b**. The tumors were collected, and the cells were stained with $CD45^+CD3e^+CD20^+PI^-$ and gated from total tumor cells.

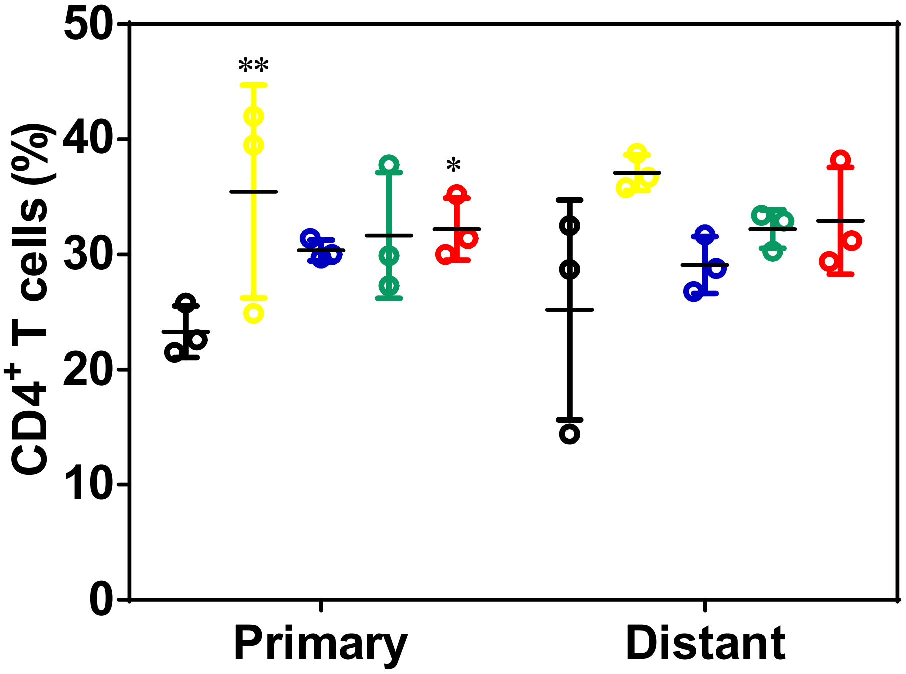

Supplementary Figure 22 Percentages of $CD4^+$ T cells in lymph nodes of MC38 tumor-bearing mice treated as described in **Figure 6a and 6b**. The lymph nodes were collected, and the cells were stained with $CD45^+CD3e^+CD4^+PI^-$ and gated from total leukocyte cells.

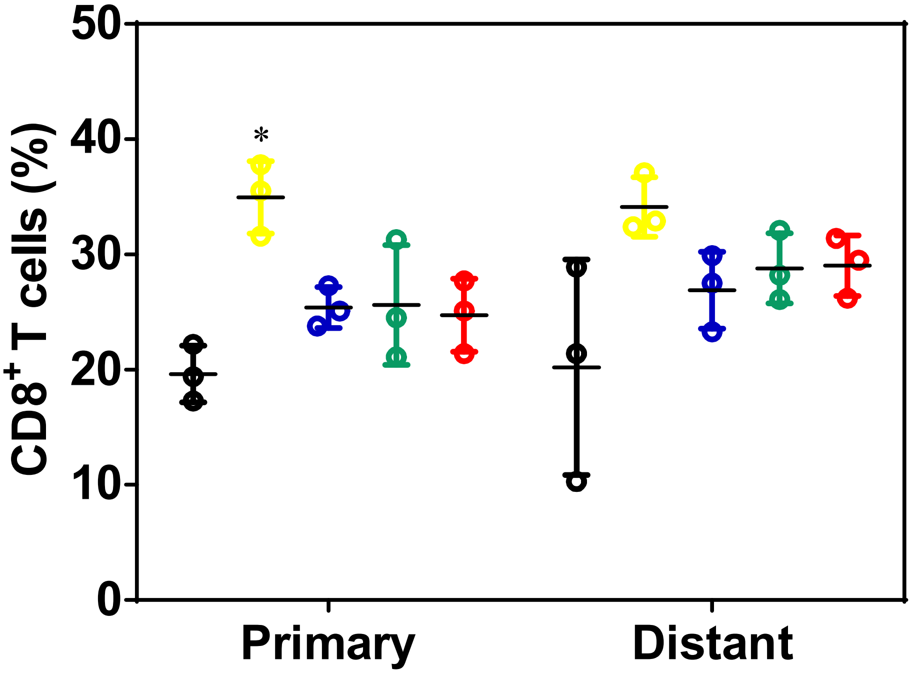

Supplementary Figure 23 Percentages of tumor-infiltrating $CD8^{+}$ T cells in lymph nodes of the MC38 tumor-bearing mice treated as described in **Figures 6a and 6b**. The lymph nodes were collected, and the cells were stained with $CD45^{+}CD3e^{+}CD8^{+}PI^{-}$ and gated from total leukocyte cells.

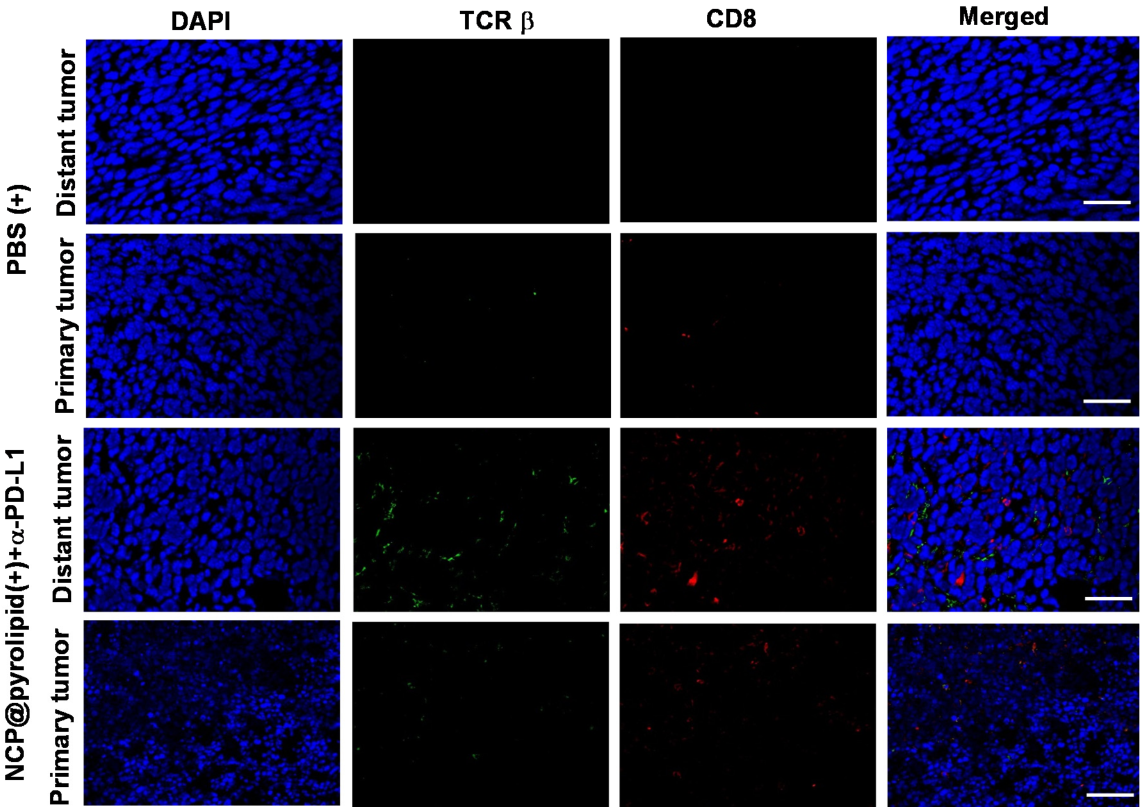

Supplementary Figure 24 Induvial channels of CLSM images of tumors after immunofluorescence staining. Bar = 50 μm.

Supplementary Tables

Supplementary Table 1 Apoptotic percentage of CT26 and HT29 cells treated with PBS, free oxaliplatin, NCP, NCP@pyrolipid, and porphysome under light and dark after a 24-h incubation

	**CT26**		**HT29**	
	**irradiation**	**dark**	**irradiation**	**dark**
PBS	0.08%	0.82%	0.04%	0.15%
Oxaliplatin	26.00%	21.40%	25.19%	25.27%
NCP	26.61%	27.23%	26.14%	27.59%
NCP@pyrolipid	35.77%	24.17%	43.10%	17.05%
Porphysome	0.20%	0.99%	0.01%	0.00%

Supplementary Methods

Materials, cell lines, and animals. All starting materials were purchased from Sigma-Aldrich and Fisher (USA) unless otherwise noted and used without further purification. 1,2-dioleoyl-*sn*-glycero-3-phosphate (DOPA), 1,2-distearoyl-*sn*-glycero-3-phosphocholine (DSPC), cholesterol, and 1,2-distearoyl-*sn*-glycero-3-phosphoethanolamine-N-[amino(polyethylene glycol)2000] (DSPE-PEG2k) were purchased from Avanti Polar Lipids (USA). Pyrolipid was synthesized in the lab as previously reported.

Porphysome synthesis. Porphysomes were formed using the protocol reported by Zheng and co-workers previously. [1-4] The lipid film consists of 55 mol% of pyrolipid, 40 mol% of cholesterol, and 5 mol% of DSPE-PEG2k. Lipid films were dried under a gentle stream of nitrogen gas and the residual solvent was removed under vacuum for 4 h. The dried lipids were kept at -20 °C prior to use. To prepare porphysomes, the lipid film was rehydrated with PBS and extruded through a polycarbonate membrane (pore size=0.1 μm, Avanti Polar Lipids) 10 times. The size of the particles was measured by DLS and the pyrolipid concentration was determined with a UV-Vis spectrophotometer (UV-2401PC, Shimadzu, Japan) and adjusted to 1 $mg \cdot mL^{-1}$ before use.

Singlet oxygen generation of NCP@pyrolipid. We used singlet oxygen sensor green (SOSG) reagent (Life Technologies, USA) to detect singlet oxygen generated by NCP@pyrolipid. After lipid coating, NCP@pyrolipid was centrifuged at 13,000 rpm for 30 min. The supernatant containing free lipid, liposome, and porphysome was discarded,

and the pellet was resuspended with PBS. Five microliters of freshly prepared SOSG solution in methanol (5 mM) was mixed with 2 mL of NCP@pyrolipid intact in PBS or disrupted with 0.5% Triton X-100. Porphysome with the addition of 0.5% Triton X-100 at the same pyrolipid concentration as NCP@pyrolipid served as a control. Samples were treated with an LED with a wavelength of 670 nm and energy irradiance of 100 mW/cm^2 for 10 s, 20 s, 30 s, 40 s, 50 s, 100 s, and 250 s, and SOSG fluorescence was measured by exciting at 504 nm and emission at 525 nm. There was no pyrolipid fluorescence contribution within this emission window.

Oxaliplatin release from NCP@pyrolipid. *In vitro* release profiles of oxaliplatin from DOPA-NCP and NCP@pyrolipid were performed in 200 mL PBS buffer at 37 ^oC and pH 7.4. DOPA-NCP or NCP@pyrolipid (0.75 mg) was sealed in a dialysis bag (MWCO 100,000), and immersed in 200 mL of PBS buffer with stirring. Periodically, 1 mL aliquots of solution were taken from the solution, and a fresh 1 mL of buffer solution was added to the beaker. The removed aliquot was digested in nitric acid and analyzed by ICP-MS for Pt.

Cellular uptake of NCP@pyrolipid. CT26 cells were seeded on 6-well plates at 5×10^5 cells/well and incubated for 24 h. NCP@pyrolipid, NCP, free cisplatin, or porphysome was added to the cells at a cisplatin dose of 5 μM or pyrolipid dose of 1.6 μM, respectively. After incubating for 1, 2, 4, and 24 h, CT26 cells were collected, washed with PBS three times, and counted with a hemocytometer. The cells were centrifuged at 3,000 rpm for 5 min and the cell pellet was digested with 500 μL of concentrated nitric acid. After 24 h, the

digestion was diluted with water and subjected to ICP-MS to determine the Pt concentration. Results were expressed as the amount of oxaliplatin (pmol) per 10^5 cells. The amount of pyrolipid being taken up by the cells was quantified with a spectrofluorophotometer (RF-5301PC, Shimadzu, Japan). After incubating with NCP@pyrolipid for 1, 2, 4, and 24 h, CT26 cells were washed with PBS three times, counted with a hemocytometer, and lysed with 0.5% (w/v) SDS (pH 8.0). The fluorescence intensity of pyrolipid was determined by fluorimetry (λ_{ex}=427 nm, λ_{em}=675 nm). Results were expressed as the amount of pyrolipid (pmol) per 10^5 cells.

Anti-tumor vaccination. A total of 5×10^5 CT26 cells treated with PBS or NCP@pyrolipid and light irradiation were inoculated subcutaneously into the right flank region of 6-week-old male BALB/c mice. One week later, these mice were rechallenged by injecting 1×10^5 CT26 cells into the contralateral flank. All the mice were sacrificed when the size of the right tumors in the PBS group exceeded 2 cm^3.

Supplementary References

1. Jin, C.S., Cui, L., Wang, F., Chen, J. & Zheng, G. Targeting-triggered porphysome nanostructure disruption for activatable photodynamic therapy. *Adv Healthc Mater* **3**, 1240-1249 (2014).
2. Lovell, J.F. et al. Enzymatic Regioselection for the Synthesis and Biodegradation of Porphysome Nanovesicles. *Angew Chem Int Edit* **51**, 2429-2433 (2012).
3. Lovell, J.F. et al. Porphysome nanovesicles generated by porphyrin bilayers for use as multimodal biophotonic contrast agents. *Nat Mater* **10**, 324-332 (2011).

4. Carter, K.A. et al. Porphyrin-phospholipid liposomes permeabilized by near-infrared light. *Nat Commun* **5** (2014).
